# Fabrication of Small-Pixel CdZnTe Sensors and Characterization with X-rays

**DOI:** 10.3390/s21092932

**Published:** 2021-04-22

**Authors:** Stergios Tsigaridas, Silvia Zanettini, Manuele Bettelli, Nicola Sarzi Amadè, Davide Calestani, Cyril Ponchut, Andrea Zappettini

**Affiliations:** 1 European Synchrotron Radiation Facility (ESRF), 71 Avenue des Martyrs, F-38043 Grenoble, France; ponchut@esrf.fr; 2 Due2lab s.r.l., via Paolo Borsellino 2, 42019 Scandiano, Italy; zanettini@due2lab.com; 3 IMEM-CNR, Istituto Materiali per l’Elettronica e il Magnetismo, Consiglio Nazionale delle Ricerche, Parco Area delle Scienze 37/A, 43124 Parma, Italy; manuele.bettelli@imem.cnr.it (M.B.); nicola.sarziamade@imem.cnr.it (N.S.A.); davide.calestani@imem.cnr.it (D.C.); andrea.zappettini@imem.cnr.it (A.Z.)

**Keywords:** cadmium zinc telluride (CdZnTe), vertical Bridgman, high-Z sensors, pixel detectors, X-ray detectors, Timepix

## Abstract

Over the past few years, sensors made from high-Z compound semiconductors have attracted quite some attention for use in applications which require the direct detection of X-rays in the energy range 30–100 keV. One of the candidate materials with promising properties is cadmium zinc telluride (CdZnTe). In the context of this article, we have developed pixelated sensors from CdZnTe crystals grown by Boron oxide encapsulated vertical Bridgman technique. We demonstrate the successful fabrication of CdZnTe pixel sensors with a fine pitch of 55 m and thickness of 1 mm and 2 mm. The sensors were bonded on Timepix readout chips to evaluate their response to X-rays provided by conventional sources. Despite the issues related to single-chip fabrication procedure, reasonable uniformity was achieved along with low leakage current values at room temperature. In addition, the sensors show stable performance over time at moderate incoming fluxes, below $10^{6}$ photons $mm^{-2}s^{-1}$.

## 1. Introduction

Different materials have been evaluated in recent years for hybrid pixel radiation detectors in search of the best choice for X-ray imaging in the 30–100 keV range. The detection of radiation with energy above 20 keV requires the exploitation of high-Z sensing materials with enhanced absorption efficiency [1], since silicon detectors are almost transparent in this energy range. The principal candidates are presently gallium arsenide (GaAs) [2], cadmium telluride (CdTe) [3,4], and cadmium zinc telluride (CdZnTe), with typical thickness up to a few millimeters.

CdZnTe is a wide band gap semiconductor with high detection efficiency (for instance, 2 mm-thick CdZnTe stops 85% of X-rays at 100 keV) which can operate at room temperature with optimal performance [5]. CdZnTe is less prone to self-polarization, which instead affects CdTe [6,7]. Thanks to its technological advantages, presently CdZnTe applications cover homeland security [8], industrial non-destructive tests and quality control [9], aerospace [10] and medical imaging [11,12].

The basic requirements of the semiconductors for hybrid pixel detectors for X-ray imaging are stability over time and uniformity of crystals properties and detector performance, or in other words, absence of inhomogeneities bigger than the detector spatial resolution [13]. One of the main causes of detector performance degradation in CdTe and CdZnTe is the presence of Tellurium inclusions, which are known to cause losses in charge collection [14]. To ensure uniformity in detector panel performance, it is essential to analyze the relation between bulk defects distribution and homogeneity of X-ray response.

In the context of this article, small-pixel CdZnTe sensors hybridized with Timepix ASIC have been tested to respond to the new sensor requirements for the Extremely Brilliant Source (EBS) upgrade of the European Synchrotron Radiation Facility (ESRF). Prior tests of X-ray imaging with fine-pixel pitch CdZnTe detectors have been carried out with Timepix ASIC [15] (55 and 110 μm pitch) and with HEXITEC ASIC [16,17], (250 μm pitch). Some of these recent contributions make use of a special CdZnTe grown by Redlen Technologies and optimized for applications with high photon fluxes. This soon attracted the attention for applications in new generation synchrotrons [18] or X-ray Free Electron Lasers (XFEL) [19].

The aim of this article is to test the performance of fine-pixel pitch sensors made with CdZnTe grown at IMEM-CNR and analyze it in relation to Te inclusions defect distribution acquired by extensive infrared scan. The sensors have been fabricated by IMEM-CNR in collaboration with Due2lab srl, and successively bump-bonded by third parties to Timepix electronics. X-ray characterization is carried out with conventional X-ray sources available at the European Synchrotron Radiation Facility (ESRF).

CdZnTe grown at IMEM-CNR (Parma, Italy) by Boron oxide encapsulated vertical Bridgman technique has already demonstrated optimal performance, both with a single-pixel contact geometry [20] and sub-millimeter pixel geometry (500 and 250 μm pitch) [21]. The excellent spectroscopic performance of this material and the absence of radiation-induced polarization effects up to fluxes of $2.2\times 10^{6}$ photons $mm^{-2}s^{-1}$ motivated the decision to test CdZnTe grown at IMEM-CNR for synchrotron application. CdZnTe sensors are indeed capable of delivering both high detection count rates and high-resolution spectroscopic performance, although it is challenging to achieve both attributes simultaneously [12]. It is crucial to understand advantages and limitations of this type of sensor with the aim to find out an optimal detector design for the beamlines of the upgraded ESRF.

## 2. Materials and Methods

### 2.1. Sensor Fabrication

Sensors are fabricated with CdZnTe material grown by the Boron Oxide Encapsulated Vertical Bridgman Technique at IMEM-CNR [22]. The polycrystalline charge was obtained by direct synthesis of the pure elements [23] and thermally treated before growth in such a way to reduce the off-stoichiometry [24]. The ingots have been cut into wafers along the growth axis to avoid variation of the Zn content due to Zn segregation. The monocrystals inside the wafers have been tracked down by means of IR microscopy and successively cut with a diamond saw to the final requested sensor dimensions, i.e., 14.5×14.5×2 $mm^{3}$ and 14.5×14.5×1 $mm^{3}$. For research purposes, two different CdZnTe thicknesses have indeed been investigated, 1 mm and 2 mm. The two sensors are made from two diverse ingots that slightly differ in Te inclusions distribution, as explained in Section 3.1. Due to a small alignment error during the cut, the 2-mm thick sensor contains a crystal grain boundary close to an edge.

**Figure 1:**
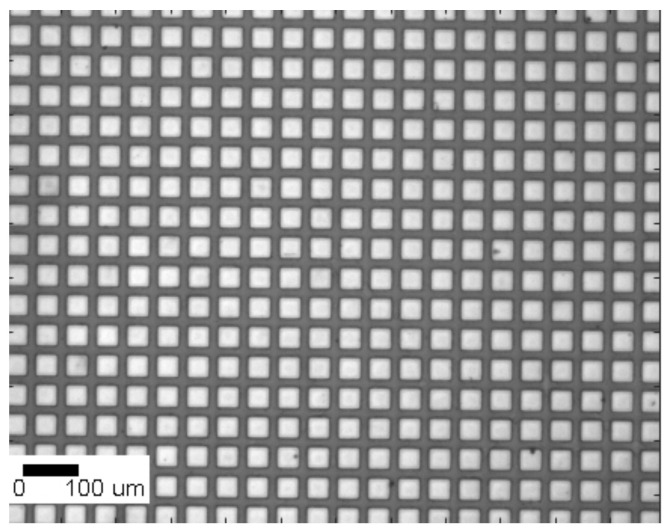
Optical microscope image showing the pixelated anode after all steps of the contact deposition process.

Before contact deposition, surfaces were lapped by means of SiC-abrasive paper (grit size P1000 and P2500) and then chemo-polished. Au contacts were deposited on both cathode and anode surfaces by chemical electroless deposition from methanol solution [25]. In order to completely remove surface oxidation, a short Br-based etching was carried out right before Au deposition. A matrix of 256×256 pixels (55 μm pitch) with guard-ring (40 μm) is patterned by photolithography of the anode surface: first, the pixel area (40×40 $\mu m^{2}$) is covered with positive photoresist, and successively the metal in the inter-pixel gap (15 μm) is etched with a Br${}_{2}$ solution. Figure 1 shows a close look of the pixel anode.

This process leads to an inter-pixel dark current satisfactorily low and no further passivation step is needed. The guard-ring external dimensions are 14.175×14.175 $mm^{2}$, thus the blind edge around the active area of the sensor is only 160 μm. No dicing has been carried out at the end of the processing.

The two sensors have been fabricated individually, and following identical procedures. It is worth noting that single-sensor fabrication presents some criticalities with respect to wafer-level processing; these are mostly related to sensor handling, which occasionally causes small defects on the sensor edge, such as scratches and edge chips.

### 2.2. Detector Assembly

**Figure 2:**
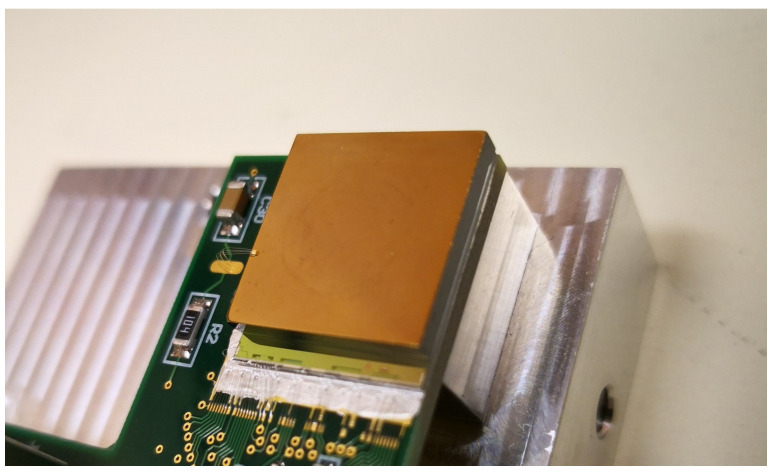
A photograph showing a CdZnTe sensor bump-bonded on a Timepix chip. The hybrid structure is mounted on custom-designed printed circuit board.

The CdZnTe sensors after the fabrication and the electrical characterization were coupled to Timepix chips [26] with a low temperature flip-chip process. The hybrid structure consisting of the CdZnTe sensor and the Timepix chip was then mounted on a custom-designed printed circuit board (PCB) compatible with the MAXIPIX readout system [27]. The chip was connected to the PCB by means of wire-bonding. For the bias voltage contact, a few thin wires (see Figure 2) were used to connect the dedicated pad on the PCB with the cathode electrode. Finally, the detector was placed in metallic housing, where it is protected from ambient light. The measurements presented in this article were performed at room temperature.

### 2.3. 3D Inclusion Mapping

Regardless of the crystal growth technique, CdZnTe ingots are always characterized by a secondary phase of Te inclusions to a greater or lesser extent. These defects act as recombination centers which locally degrade the charge collection efficiency and, hence, the spectroscopic performance of the final device [28]. Bolotnikov et al. reports that the detector performance is correlated with inclusion distribution [29]. The impact is even more important in pixelated detectors whose pitch is of the same order of magnitude as the larger inclusions (>100 μm) because their presence may completely hamper the functioning of one or more pixels, depending on their positioning along the crystal thickness. Therefore, before depositing gold contacts, we performed a thorough 3-D mapping of the whole crystals by means of IR microscopy using the setup as described in Zambelli et al. [30].

A DMK33UP1300 IR camera (Theimagingsource) is connected to a Nikon MICROPHOTFXA microscope, and a near-infrared high-power LED peaked around 850 nm is used. This system allows scanning automatically crystals with surface dimensions up to 50×50 $mm^{2}$. Before the acquisition of the sample, the flat-field and dark-field images are acquired at the same exposure and electronic gain settings to pre-process the raw images whose intensity is subsequently equalized. During the measurements, the crystals are divided into virtual voxels with surface area equal to 512×640 $\mu m^{2}$, and several images are acquired at different focal depths of each voxel. Hereinafter, each set of stacked images acquired at different focal depths is considered to be a voxel. For each image, the 3D gradient is calculated by means of the Sobel algorithm to recognize the contours (i.e., where the gradient exceeds a predetermined threshold) and the images are converted to binary ones in which the value “1” indicates the presence of an inclusion.

Since the inclusions leave a trace in multiple images at various depths, an algorithm determines the true position and dimension by means of the wavelet analysis tool. Basically, the image is decomposed to extract the elements of the image that are in focus. A sub-volume is thus identified for every inclusion. The algorithm also determines if other small inclusions are contained in the macro binary volume, to avoid a possible underestimation of total Te volume. For each previously identified sub-volume, the center of gravity and real size of each inclusion are identified. Finally, the map of the whole crystal is obtained by merging the results for each voxel. For inclusions with a diameter greater than 1 μm, this method can be considered reliable. Further details are given in the related patent [31].

A DMK33UP1300 IR camera (Theimagingsource) is connected to a Nikon MICROPHOT-FXA microscope, and a near-infrared high-power LED peaked around 850 nm is used. This system allows scanning automatically crystals with surface dimensions up to 50×50 $mm^{2}$. During the measurements, the crystals were divided into virtual voxels with surface area equal to 512×640 $\mu m^{2}$, and several images are acquired at different focal depths of each voxel. Successively, an algorithm processed each voxel (which is practically a package of stacked images) to extract the 3-D coordinates and dimensions of the detected inclusions. Finally, the map of the whole crystal was obtained by merging the results for each voxel.

### 2.4. 3D X-ray Detector Response

Once the detectors have been bonded, the response of the sensors to illumination with X-rays was measured at ESRF, by using laboratory X-ray sources. Below we briefly describe the main specifications of each source and the tests performed. The configuration settings of these sources, as used in this article, are summarised in Table 1.

**Source 1**  A conventional laboratory X-ray generator with exchangeable anodes, able to provide a broad X-ray beam. This source was used for the energy calibration of detector modules. In addition, this source is suitable for basic imaging tests which require uniform illumination and a moderate incoming flux.
**Source 2**  A conventional laboratory X-ray generator featuring a fixed copper (Cu) anode, with the possibility to focus the beam. The beam is initially shaped by focusing optics and then is passing through a set of collimating slits. The final collimation is performed by a circular pinhole that allows configuration of the beam spot size down to 10 μm. This source is used to perform sensitivity mappings of the sensors at the sub-pixel level or to study the charge transport properties of the sensor material.

## 3. Results and Discussion

### 3.1. IR Mapping

**Figure 3:**
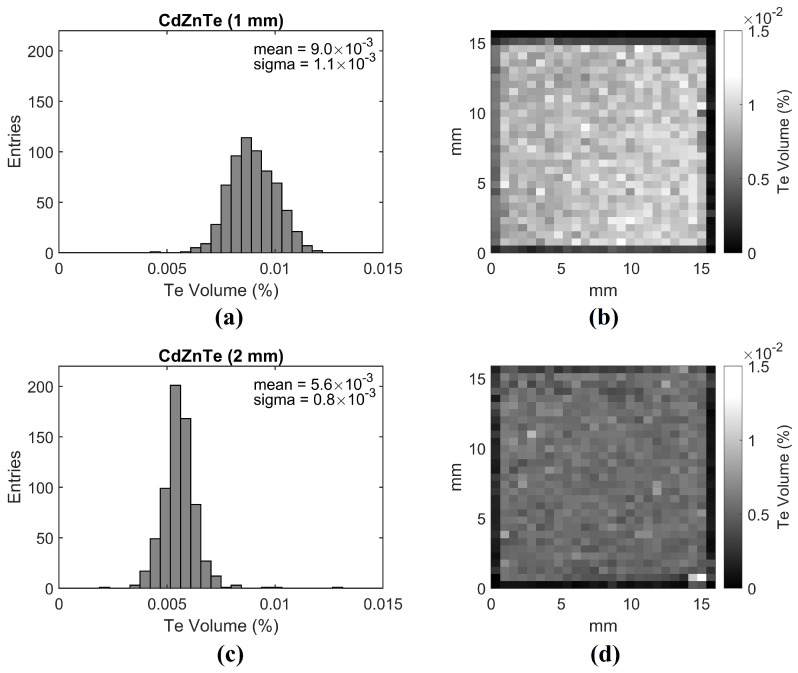
Histograms of the percent fraction occupied by Te inclusions in each voxel of the crystal volume, for the 1 mm (**a**) and 2 mm-thick (**c**) detectors, respectively. Corresponding x-y map of the crystal showing the uniformity for the 1 mm-thick (**b**) and 2 mm-thick (**d**) detectors. The maps are affected by edge effects because the external rows of voxels are only partially filled by the crystal volume; these external voxels have been excluded from the histograms (**a**,**c**) to avoid artifacts.

IR inspection was performed on the bare crystals after polishing and before contact deposition; the polished surfaces ensured the correct transmission of IR light. Figure 3a,c show the histogram of the percent fraction occupied by Te inclusions in each voxel of the crystal volume, for the 1 mm (a) and 2 mm-thick (c) detectors, respectively. Figure 3b,d represent the spatial distribution of the Te fraction. The average percentage of Te content is $\left(9.0\pm 1.1\right)\times 10^{-3}$ (%) for 1 mm-thick detector and $\left(5.6\pm 0.8\right)\times 10^{-3}$ (%) for 2 mm-thick detectors. Nevertheless, both detectors show a good homogeneity in terms of Te content (%).

**Figure 4:**
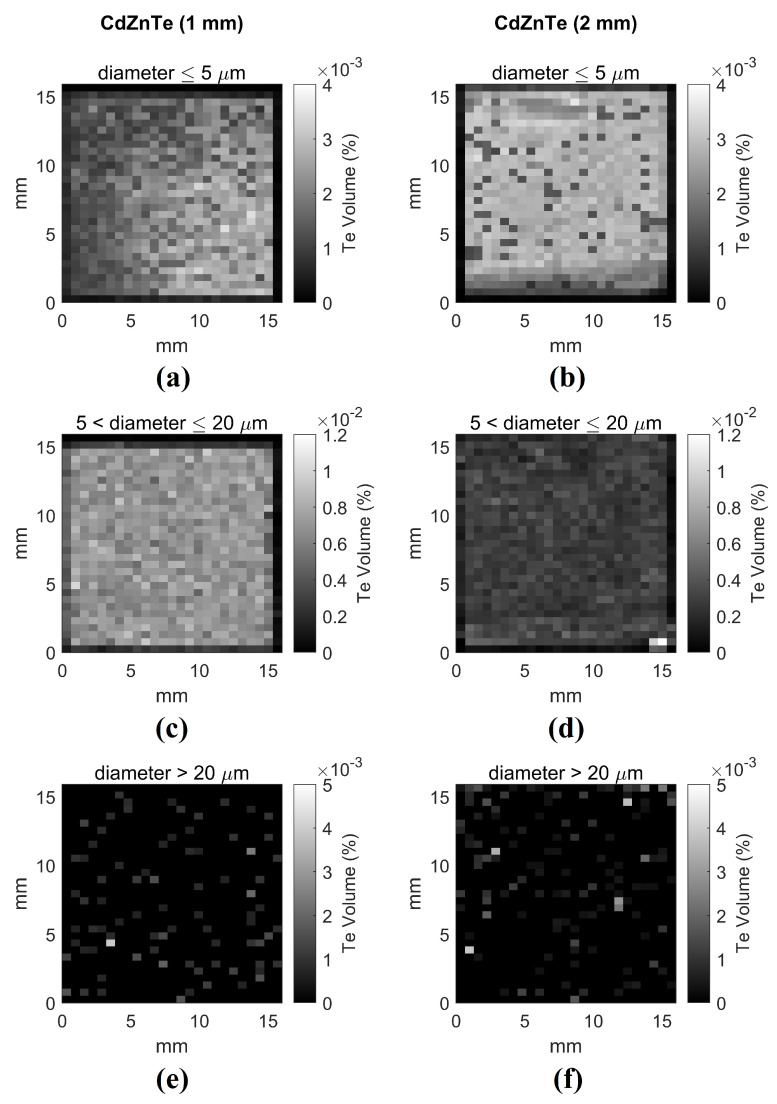
2D histograms showing the fraction in percentage of the volume occupied by Te inclusions with diameter smaller than 5 μm (**a**,**b**), ranging from 5 to 20 (**c**,**d**) and greater than 20 (**e**,**f**) for the 1 mm-thick (**left**) and 2 mm-thick (**right**) detectors, respectively.

The distribution of Te inclusions can be also used as an indication of other structural defects of the crystal. Grain borders and dislocations tend to accumulate inclusions in their surroundings. If we focus the attention on the smallest inclusions, with diameters up to 5 μm, some inhomogeneities arise in both sensors. A broad concentration gradient can be observed across the whole crystal of the 1 mm-thick detector, as shown in Figure 4a. This characteristic is probably due to the Vertical Bridgman growth technique, which is often characterized by radial differences near the edge of the ingot: the small inclusions concentration has been altered by the temperature gradient in this region. On the other hand, the 2D histogram of 2 mm-thick detector shows a slightly oblique linear edge in the lower part of Figure 4b, which divides a light and a dark region. This feature is an indication of a twin border, which usually manifests itself in groups of 3 to 6 parallel twin planes whose interfaces lie exclusively in the {111} plane [32]. Moreover, the 2 mm-thick detectors show a high concentration of inclusions with a diameter between 5 and 20 μm in the bottom right corner of Figure 4d: this suggests the presence of a grain border. The interruption of crystal periodicity has a significant impact on the transport properties of the material and thus it also appears on the final X-ray images, as shown in the next sections. No evident patterns were observed in the maps of inclusions with diameters greater than 20 μm (Figure 4e,f).

### 3.2. Visual Inspection

By means of an automatized system of visual inspection, we have counted the number of disqualified pixels of each detector after photolithography and before bump-bonding. A pixel is considered disqualified if it is short-circuited with the neighboring pixel (in this case we count 2 pixels disqualified) or damaged, thus susceptible of improper charge collection, for example when gold deposition is defective or persistent organic residues are covering the pixel metal contact. The 2-mm thick sensor presents 0.15% of disqualified pixels (95 over 65,536), while the 1 mm-thick sensor has 0.18% of disqualified pixels (118 over 65,536).

### 3.3. Electrical Characterization

#### 3.3.1. Before Interconnection

Both sensors were tested electrically before bump-bonding to verify that the pixel dark leakage current is low enough. IV-characteristics of a few random pixels were measured with a probe station, by polarizing the cathode negatively up to −500 $V\;mm^{-1}$, while pixel and guard-ring were grounded by connecting them with two probe tips. The probe station is positioned inside a Faraday cage and IV curves are taken after a few minutes the sensor is placed in the dark. We obtain an average dark leakage current value around 1 nA for both sensors, which may appear excessively high for the correct sensor functioning. However, this current value includes the contribution to the current of a large fraction of the sensor anodic surface, due to the fact that neighboring pixels were not grounded. We deduce that pre-bonding electrical characterization of fine-pixel pitch sensors is useful to roughly estimate the goodness of the metal contact, but not indicative of the real dark current value under operation: it is reasonable to expect a significantly lower value once the sensor is bonded and the pixel matrix fully connected.

#### 3.3.2. After Interconnection

After the bump-bonding procedure the IV-characteristic of the sensor is measured again. The bias voltage to the sensor is provided through a contact to the backside electrode, as shown in Figure 2. In order to generate the high voltage, we use an external power supply which is controlled remotely. The IV-characteristic for each sensor is then extracted using as described in [15].

**Figure 5:**
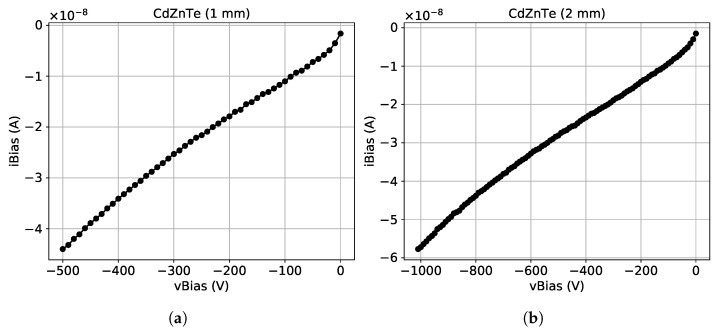
Measured IV-characteristics at room temperature for CdZnTe sensors with 55 μm pixel pitch and a thickness of (**a**) 1 mm and (**b**) 2 mm.

As described above, the sensor pixel array is surrounded by a guard-ring. It is worth mentioning that for both sensors the connection of the guard-ring was not established. The measured current values in this case are assumed to be the sum of the leakage currents of each pixel. During this measurement, the Timepix chip is powered on while the sensor is at room temperature and protected from ambient light. The measured IV curves for the 1 mm thick and the 2 mm thick sensor are shown in Figure 5a,b, respectively.

The maximum value of the bias voltage for both sensors corresponds to an electric field strength of 500 $V\;mm^{-1}$. This is the typical setting that we use as operating point for CdZnTe sensors. At this setting the total leakage current measured is −44 nA for the 1 mm thick sensor and −57 nA for the 2 mm thick one, which correspond to an equivalent leakage current for a single pixel of 0.67 pA and 0.87 pA respectively. In the 2 mm thick sensor, due to its larger thickness, the thermally generated leakage current is expected to be higher, which is in line with the values observed. In both cases, the values observed are much lower than the leakage current compensation limit of the dedicated circuitry within a pixel of the Timepix chip.

### 3.4. Characterization with X-rays

To characterize the performance of the modules with X-rays we proceed first with the threshold equalization and the energy calibration. The pixel-to-pixel threshold variations are adjusted using the noise as described in [26]. Then the energy calibration is performed using the characteristic X-rays from Source 1. In the next subsections we present results obtained concerning the sensor uniformity at the macroscopic and the microscopic level, the charge transport properties and the stability over time.

#### 3.4.1. Flood Images

**Figure 6:**
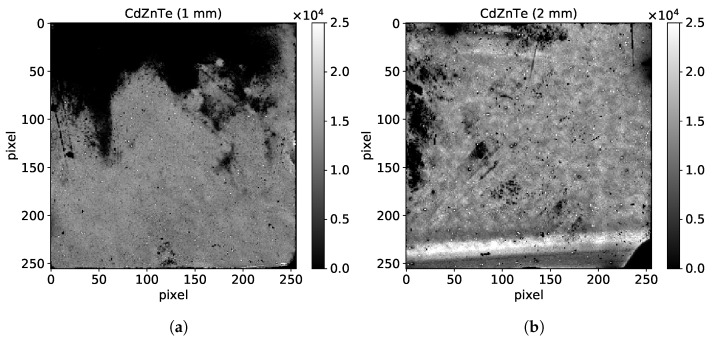
Raw flood images recorded with CdZnTe sensors of 55 μm pixel pitch and a thickness of (**a**) 1 mm and (**b**) 2 mm. The energy threshold was set at half of the characteristic energy of Source 1.

Both sensors were illuminated using the wide uniform beam provided by Source 1 to obtain flood images, with the Timepix chip operated in counting mode. After the equalization and the calibration procedure, pixels with an excessive number of counts are considered noisy and therefore are masked. Figure 6 shows the raw flood images obtained with both sensors.

The 1 mm thick sensor contains a large defective area at its top side, which was not detectable with standard visual inspection. Within this area there are mostly non-responsive pixels and noisy pixels that had to be masked. However, there is also a small fraction of responsive pixels which record a very low number of photons and appear as dark. This behavior can be attributed to surface defects occurring during the sensor fabrication procedure. Such surface defects are also the origin of other dark spots observed within the sensor. In addition, we observe defects at the edges and the corners of the sensor related to single-chip processing, as described in in Section 2.1. Nevertheless, we observe a large area over the sensor with reasonable uniformity and pixels behaving normally.

On the other hand, the 2 mm thick sensor contains several areas with reasonable uniformity which denotes the absence of bulk defects, except in the low right corner which appears black as a result of the presence of a grain boundary. The uniform areas though reveal a rougher structure with respect to the 1 mm thick case which is related to the fact that this sensor comes from a different crystal ingot. We also observe dark spots and similar edge effects as in the 1 mm thick sensor, which could be mitigated by grounding of the guard-ring. Moreover, we observe several bright spots, a few pixels wide, which are surrounded by dark regions. Similar surface defects were observed in the past with CdTe sensors [7] and they typically alter the electric field locally, inducing distortions. The thick line pattern observed at the bottom side is due to the twin border identified with the IR mapping. Crystal twinning does not completely hinder the functionality of the interested region in contrast to grain boundaries. However, the resistivity is locally altered and consequently the local electric field. As a result the pixels within this region record an excessive count rate and therefore this region appears as brighter.

#### 3.4.2. Sub-Pixel Mapping

Using the micro-focused beam provided by Source 2 we can perform sensitivity maps of the sensor at the sub-pixel level. In this way we could extract more information concerning the uniformity of the sensor material. To do so, we mount the detector on a set of precision translation stages, with the incident beam direction being perpendicular to translation directions. The detector is facing the beam while we align the beam at the center of a chosen pixel and scan its surrounding area. Using a scan step of 5 μm, much smaller than the actual pixel size, we record a single image for each scan point.

**Figure 7:**
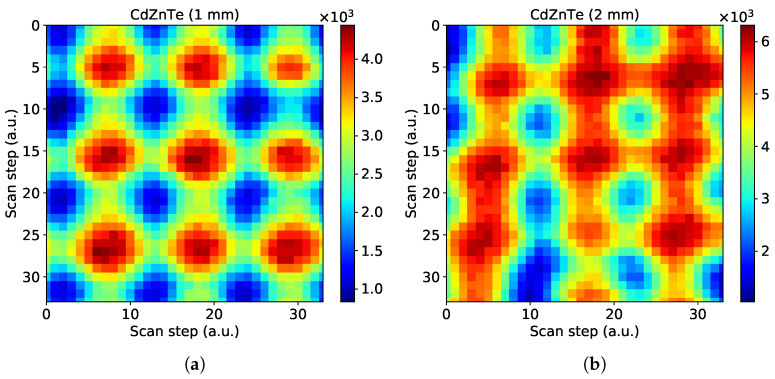
Sensitivity maps of a 3 × 3-pixel area within CdZnTe sensors with 55 μm pixel pitch and a thickness of (**a**) 1 mm and (**b**) 2 mm. The raster scans performed with a step of 5 μm with the energy threshold set at 5 keV.

Figure 7 shows reconstructed sensitivity maps of 3 × 3 pixel areas obtained with both sensors. The energy threshold for this measurement was set at 5 keV, slightly above the half of the characteristic energy of Source 2 to minimize the charge sharing between pixels. For both sensors we see that the effective pixel shape is regular with slight variations in the size, which is typical for small-pixel sensors made from compound semiconductors. In the 2 mm sensor, as expected the charge sharing is higher due to the larger charge diffusion, taking into account the sensor thickness.

**Figure 8:**
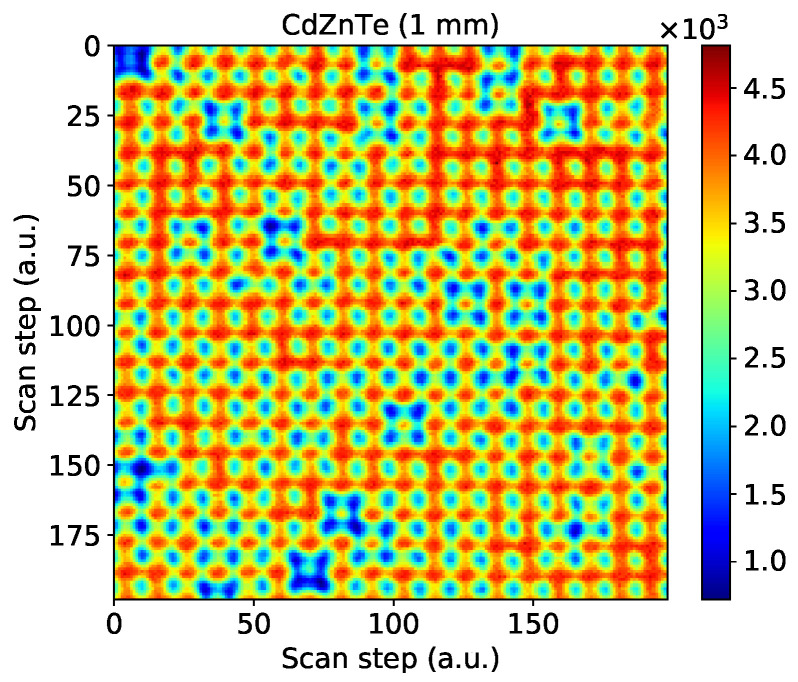
Sensitivity maps of a pixel area covering 1 $mm^{2}$ within a CdZnTe sensor with 55 μm pixel pitch and a thickness of 1 mm. The raster scans performed with a step of 5 μm with the energy threshold set at 4.5 keV.

To check the uniformity of the material on a larger extent, we perform a raster scan over a 1 $mm^{2}$ area using the 1 mm thick sensor. The scan area is chosen carefully to avoid significant sensor defects which induce distortions and variations of the effective pixel shape. The result of the scan is shown in Figure 8. We still observe a few pixels which seems to be smaller in size therefore are less efficient. Overall, the uniformity in this selected area is high, without large variations and is indicative of the sensor quality that can be achieved with this fabrication process.

#### 3.4.3. Mobility-Lifetime Product ($\mu_{e}\tau_{e}$)

**Figure 9:**
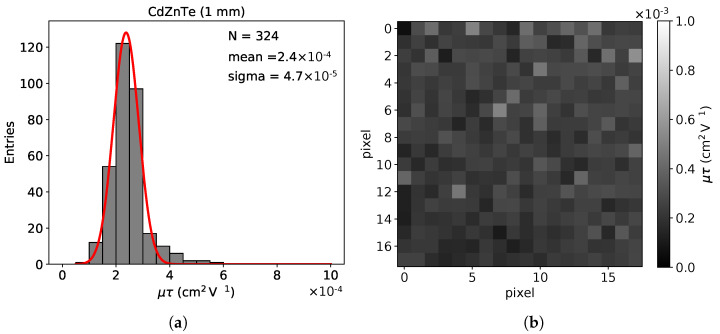
(**a**) The measured values of the mobility-lifetime product of electrons $\mu_{e}\tau_{e}$, for all the pixels within a 1 $mm^{2}$ area of the 1 mm thick CdZnTe sensor. (**b**) The corresponding 2D histogram showing the uniformity.

The uniformity of a sensor can be also expressed in terms of the charge transport properties. In our case, we study the charge transport properties through the mobility-lifetime product of the electrons $\mu_{e}\tau_{e}$. During this measurement the Timepix chip was operated in the time-over-threshold (ToT) mode [26]. The threshold was set at the minimum value ∼2.5 keV, making sure that we are above the noise level. The $\mu_{e}\tau_{e}$ of single pixels within a 1 $mm^{2}$ sensor area was measured using the method described in [33]. The measurement is based on the fitting of the experimental data using the Hecht’s equation, modified to take into account the small pixel effect [33]. The results are summarized in Figure 9.

In previously published articles using similar CdZnTe sensors [20] the values reported were in the range of the $\mu_{e}\tau_{e}=\left(6-10\right)\;\times 10^{-4}$ $cm^{2}V^{-1}$. However, these values were obtained with planar sensor and therefore no small pixel effect. This along with the effect of the energy threshold led to the lower $\mu_{e}\tau_{e}$ value that we observe in our case. Nevertheless, the measured values for single pixels over the 1 $mm^{2}$ area of the sensor without defects are reasonably uniform as expected.

#### 3.4.4. Stability

**Figure 10:**
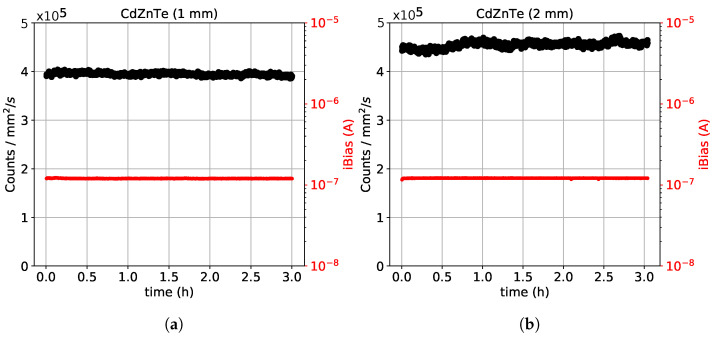
Monitoring of the count rate (in black) and the total leakage current (in red) over irradiation time for CdZnTe sensors of 55 μm pixel pitch and a thickness of (**a**) 1 mm and (**b**) 2 mm. The energy threshold was set at half of the characteristic energy of Source 1.

Source 1 was used to perform stability tests over irradiation time for both sensors. For this test the detector was mounted at about 1.5 m for the source. The detector was operated in counting mode with an energy threshold of 8.5 keV. This corresponds to the half of the characteristic X-ray energy of Source 1, so that the charge sharing contribution to the count rate is reduced to a small fraction. Then we monitor the measured count rate and the total leakage current over a course of 3 h. The results of the stability test (performed at room temperature) for both sensors are summarized in Figure 10.

From Figure 10 we observe that both sensors show a stable performance over time. The total leakage current of both sensors is identical as expected and remains stable at about 0.12 μA. This corresponds at a pixel equivalent leakage current of 1.83 pA which remains within the compensation limits of the Timepix chip. With the 1 mm thick sensor we measure a photon flux of about $4\times 10^{5}$ photons $mm^{-2}s^{-1}$, consistent with the expected output flux of Source 1 at the specific distance where the detector mounted. With the 2 mm thick sensor we observe a slightly higher measured flux of about $4.5\times 10^{5}$ photons $mm^{-2}s^{-1}$, with small oscillations. This behavior is attributed to a possible slight difference in the actual energy threshold setting and is not related to the intrinsic quantum efficiency of the sensor.

At the moderate incoming flux provided by Source 1, the measured count rate is stable over time. We do not observe any deterioration of the count rate associated with polarization effects. At higher incoming fluxes above $10^{6}$ photons $mm^{-2}s^{-1}$ polarization effects are expected to arise. However, this material due to its spectroscopic nature is not meant for use in high flux applications and therefore in this article we do not attempt any test at higher fluxes.

## 4. Conclusions

We characterized CdZnTe pixel sensors with a fine pitch of 55 μm, using the MAXIPIX readout system. Two sensors with 1 mm and 2 mm thickness have been fabricated with CdZnTe material grown at IMEM-CNR (Parma) by Boron Oxide Encapsulated Vertical Bridgman Technique. Prior to the detector processing, the defect densities in CdZnTe crystals were characterized by infrared (IR) microscopy. Metal contacts have been deposited by Au electroless deposition from alcoholic solution, which ensures a stronger contact adhesion to CdZnTe.

The sensors bump-bonded to the ASIC provide an extremely small dark leakage current at −500 $V\;mm^{-1}$ polarization. X-ray flood images and sub-pixel raster scans at different scales reveal the presence of several areas over the sensor with reasonably homogeneous pixel sensitivity and uniformity in terms of mobility-lifetime product. The combination of IR inspection and X-ray mapping shows that Te inclusions do not affect sensor functioning nor single pixels performance, except where a crystal twin is present. We infer that low response areas in flood images are probably due to sensor surface defects or metal deposition inhomogeneities on anode and/or cathode side, but not to bulk defects.

The performance is stable over time for count rates up to 4 ×$10^{5}$ photons $mm^{-2}s^{-1}$. At higher incoming fluxes above $10^{6}$ photons $mm^{-2}s^{-1}$ polarization effects are expected to arise. However, this material due to its spectroscopic nature is not meant for use in high flux applications and therefore we do not attempt tests at higher fluxes.

Even though a non-negligible number of pixels is noisy or non-responsive, the successful fabrication of 55 μm pitch pixel sensors has been demonstrated. The possibility of CdZnTe processing at wafer-level would surely improve pixel response uniformity. Future plans include the estimation of sensor spatial resolution and tests at higher energies.

## Figures and Tables

**Table 1:** Characteristics of the X-ray sources used.

Source Features	Source 1	Source 2
Anode	Molybdenum (Mo)	Copper (Cu)
Peak voltage/Current	25 kV/25 mA	31 kV/1 mA
Filtering material	Zirconium (Zr)	Copper (Cu)
Filtering thickness	100 μm	50 μm
Characteristic X-ray energy	17 keV	8 keV

## Data Availability

Data available on request.

## Abbreviations

The following abbreviations are used in this manuscript:

GaAs	Gallium Arsenide
CdTe	Cadmium Telluride
CdZnTe	Cadmium Zinc Telluride
XFEL	X-ray Free Electron Lasers
ESRF	European Synchrotron Radiation Facility
IMEM-CNR	Istituto dei Materiali per l’Elettronica ed il Magnetismo-Consiglio Nazionale delle Ricerche
PCB	Printed Circuit Board
ASIC	Application Specific Integrated Circuit
ToT	time-over-threshold
